# Reduced conversion and readmission rates in robotic ileocecal resection for Crohn’s disease: a propensity-matched analysis of the Hugo™ RAS system

**DOI:** 10.1007/s00464-026-12775-9

**Published:** 2026-04-17

**Authors:** Tommaso Violante, Stefano Cardelli, Giacomo Calini, Marco Novelli, Matteo Rottoli

**Affiliations:** 1 Surgery of the Alimentary Tract, IRCCS Azienda Ospedaliero-Universitaria Di Bologna, Bologna, Italy; 2 Department of Medical and Surgical Sciences, Alma Mater Studiorum University of Bologna, Via Massarenti 9, 40138 Bologna, Italy; 3 Division of Colon and Rectal Surgery, Mayo Clinic, Rochester, MN USA; 4 Department of Statistical Sciences, Alma Mater Studiorum - University of Bologna, Bologna, Italy

**Keywords:** Crohn’s disease, Robotic surgery, Hugo RAS, Ileocecal resection, Conversion, Readmission

## Abstract

**Background:**

Robotic surgery is increasingly adopted in inflammatory bowel disease to address the technical limitations of conventional laparoscopy. This study aimed to compare the perioperative outcomes of robotic ileocecal resection for Crohn’s disease (CD) using the Hugo™ RAS system against laparoscopic and open approaches.

**Methods:**

Data were retrospectively collected from a prospectively maintained database of patients undergoing ileocecal resection for CD between January 2003 and June 2025 at a tertiary referral center. Patients were stratified by surgical approach: robotic, laparoscopic, or open. Multivariable regression, 1:1 and 1:4 propensity score matching (PSM), and G-computation were utilized to compare postoperative complications, readmissions, conversion rates, and length of hospital stay (LOS).

**Results:**

A total of 1392 patients were included (62 robotic, 623 laparoscopic, 707 open). The robotic approach was associated with a significantly lower rate of conversion to open surgery compared to laparoscopy (1.6% vs 15.2%; p = 0.001). After adjustment, robotic surgery remained independently associated with an 89% reduction in the odds of conversion (adjusted OR 0.11; 95% CI 0.02–0.77; p = 0.027). In the primary PSM analysis, the robotic group demonstrated a 16.1% absolute risk reduction in 30-day readmissions (p = 0.025) and a significantly lower risk of severe complications (Clavien–Dindo ≥ III) compared to laparoscopy (p = 0.037). Sensitivity analyses confirmed a statistically significant reduction in LOS for the robotic group compared to both laparoscopic (p = 0.049) and Open (p < 0.001) approaches. Adjusted operative times were comparable between robotic and laparoscopic procedures (p = 0.572).

**Conclusion:**

Robotic ileocecal resection using the Hugo™ RAS system is a safe and effective alternative to conventional techniques. It offers distinct clinical advantages, including marked reductions in conversion rates and hospital readmissions, as well as a shorter length of stay, without compromising operative efficiency.

Since its initial description in 1932 by Crohn, Ginzburg, and Oppenheimer, Crohn’s disease (CD) has been recognized as a chronic inflammatory condition that predominantly impacts the terminal ileum, although it can manifest in any part of the digestive tract [1, 2].

Surgical intervention remains a common necessity for this population; approximately 30% of patients will eventually require major abdominal surgery, with ileocolic resection being the most frequent procedure [3]. Current epidemiological evidence indicates that roughly 18% of patients undergo surgery within 5 years of diagnosis, a figure that rises to 26.2% by 10 years [4].

The introduction of laparoscopy in the early 2000s marked a significant shift in surgical management [5, 6]. This minimally invasive approach has been shown to offer distinct advantages over open surgery, including reduced hospital stays, lower complication rates, and improved cost-effectiveness, without compromising long-term recurrence-free survival [7–11].

More recently, robotic platforms have been integrated into practice to overcome specific technical constraints associated with conventional laparoscopy [12, 13]. By offering features such as tremor filtration, multi-articulated instrumentation, and high-definition 3D visualization, robotic systems allow for superior precision and gentler tissue manipulation [14, 15]. However, despite the increasing utilization of this technology in colorectal and inflammatory bowel disease surgery, there is a scarcity of data directly comparing its effectiveness against standard laparoscopic and open techniques.

To bridge this knowledge gap and contribute to the comparative evidence base in IBD, we performed a contemporary propensity score-matched analysis. This study evaluates outcomes in patients undergoing ileocolic resection for CD via robotic, laparoscopic, or open approaches, aiming to provide risk-adjusted evidence to inform surgical decision-making.

## Methods

### Study design and patient selection

Following approval from the Institutional Review Board, we analyzed data extracted from a prospectively maintained registry at a tertiary IBD referral center. The study period spanned from January 2003 to June 2025. Eligibility criteria included adult patients (aged 18 and older) with a confirmed diagnosis of CD who underwent elective primary or redo ileocecal resection. We excluded patients who required emergency surgery or those with missing perioperative data. This study is reported in accordance with the STROBE (Strengthening the Reporting of Observational Studies in Epidemiology) guidelines.

### Surgical groups

The cohort was categorized into three groups based on the initial surgical modality: open, laparoscopic, and robotic. All robotic cases were completed using the Hugo™ RAS system (Medtronic, Minneapolis, MN, USA) with a customized docking configuration. For the minimally invasive cohorts, conversion was defined as any unplanned incision or incision extension required to complete the operation. Primary outcomes were analyzed according to the intention-to-treat principle.

### Data variables and endpoints

Baseline data collection included demographics such as age, gender, and Body Mass Index (BMI). Clinical variables included disease duration, Montreal Classification (B1, B2, B3), comorbidities (Charlson Comorbidity Index), and the American Society of Anesthesiologists (ASA) physical status. We also recorded prior abdominal surgeries and preoperative medication use (systemic steroids or biologics) within 8 weeks of the procedure. To account for surgical complexity, we specifically identified patients undergoing concomitant procedures, defined as strictureplasties or additional bowel resections outside the ileocecal segment.

The primary outcomes assessed were 30-day postoperative complications (Clavien–Dindo classification), severe complications (Clavien–Dindo grade ≥ III), readmission within 30 days, and reoperation rates. Secondary outcomes included operative duration (skin to skin), conversion rates, and length of hospital stay (LOS).

### Statistical analysis

Continuous variables were expressed as medians with interquartile ranges (IQR) and compared using the Kruskal–Wallis test, while categorical data were reported as frequencies and percentages, analyzed via Chi-square or Fisher’s exact tests. To address baseline differences and potential confounders, such as age, BMI, comorbidities, disease phenotype, and concomitant procedures, we employed four distinct adjustment techniques. First, we used multivariable regression analysis to determine independent effects. Second, we conducted 1:1 nearest-neighbor Propensity Score Matching (PSM) to generate balanced sub-cohorts and estimate the Average Treatment Effect on the Treated (ATET). Third, a sensitivity analysis using 1:4 matching was performed to enhance statistical power and verify the robustness of the results given the sample size imbalance. Finally, G-Computation was applied to estimate adjusted absolute risks and means for the total cohort. Length of stay was analyzed using a Generalized Linear Model (GLM) with a Gamma distribution to accommodate the skewed nature of hospital stay data. Statistical significance was defined as a p value less than 0.05, and all calculations were executed using Stata SE 19.

## Results

### Study population and baseline characteristics

A total of 1392 patients underwent ileocecal resection for Crohn’s disease during the study period. The cohort was stratified by surgical approach, consisting of 62 (4.5%) robotic, 623 (44.8%) laparoscopic, and 707 (50.8%) open procedures. Baseline demographic analysis revealed significant heterogeneity between the groups (Table 1). Patients in the robotic group were significantly older (median 43.5 years; IQR 27.0–55.0) compared to the laparoscopic group (35.0 years) and the open group (41.0 years; p < 0.001). Despite being older, the robotic cohort presented with a significantly shorter median disease duration of 4.9 years compared to 6.3 years for the laparoscopic group and 8.7 years for the open group (p < 0.001).

**Table 1:** Baseline characteristics

Variables	Robotic (*n* = 62)	Laparoscopic (*n* = 623)	Open (*n* = 707)	*P*-value
Age (years), median [IQR]	43.5 [27.0–55.0]	35.0 [26.0–49.0]	41.0 [30.0–51.0]	< 0.001
BMI (kg/m^2^), median [IQR]	21.9 [19.4–24.5]	21.2 [18.9–23.9]	21.2 [18.8–23.7]	0.339
Disease duration (years), median [IQR]	4.9 [1.8–13.7]	6.3 [2.5–12.1]	8.7 [3.6–16.3]	< 0.001
Male gender, *n* (%)	31 (50.0%)	323 (51.9%)	471 (66.6%)	< 0.001
Comorbidities (any), *n* (%)	17 (27.4%)	165 (26.5%)	197 (27.9%)	0.852
Previous abdominal surgery, *n* (%)	19 (30.7%)	165 (26.5%)	241 (34.1%)	0.011
ASA score, *n* (%)				0.052
1	3 (4.9%)	30 (4.9%)	15 (2.2%)	
2	48 (78.7%)	506 (82.5%)	564 (80.7%)	
3	10 (16.4%)	76 (12.4%)	119 (17.0%)	
4	0 (0.0%)	1 (0.2%)	1 (0.1%)	
Biologic use, *n* (%)	26 (41.9%)	237 (38.0%)	227 (32.1%)	0.041
Steroid use, *n* (%)	11 (17.7%)	154 (24.7%)	216 (30.6%)	0.013
Concomitant procedures*, *n* (%)	3 (4.8%)	65 (10.4%)	160 (22.6%)	< 0.001

^*^Defined as any strictureplasty or resection of a bowel segment other than the terminal ileum performed during the same surgery

*ASA*, American Society of Anesthesiologists; *BMI*, body mass index

Distinct differences in disease phenotype were also observed. The open group contained the highest proportion of patients with fistulizing disease (54.0%), whereas the robotic group was predominantly composed of patients with stenotic disease (83.9%). Additionally, the prevalence of concomitant procedures (additional resections or strictureplasties) varied significantly, being lowest in the robotic group (4.8%) compared to the Laparoscopic (10.4%) and open (22.6%) groups (p < 0.001). Preoperative biologic use was highest in the robotic group at 41.9%, compared to 38.0% in the laparoscopic group and 32.1% in the open group (p = 0.041).

### Operative outcomes

Operative details are summarized in Table 2. The median operative time was significantly longer for robotic procedures (205 min; IQR 163–240) compared to laparoscopic (170 min) and open (180 min) approaches in the unadjusted analysis (p = 0.002). However, after adjustment using both multivariable regression (p = 0.865) and propensity score matching (p = 0.572), this difference was no longer statistically significant, suggesting that case complexity (including concomitant procedures) accounted for the variance.

**Table 2:** Operative details and unadjusted outcomes

Variables	Robotic (*n* = 62)	Laparoscopic (*n* = 623)	Open (*n* = 707)	*P*-value
Operative time (min), median [IQR]	205 [163–240]	170 [140–210]	180 [135–230]	0.002
Length of stay (days), median [IQR]	6.0 [5.0–7.0]	7.0 [6.0–8.0]	8.0 [7.0–9.0]	< 0.001
Conversion to open, *n* (%)	1 (1.6%)	95 (15.2%)	N/A	0.001
Stoma formation, *n* (%)	0 (0.0%)	2 (0.3%)	21 (3.0%)	< 0.001
Anastomosis: stapled, *n* (%)	62 (100.0%)	426 (68.4%)	465 (65.8%)	< 0.001

^*^Fisher’s exact test

A major finding was the rate of conversion to open surgery, which was lower in the robotic group (1.6%) compared to the laparoscopic group (15.2%; p = 0.001). In the multivariable logistic regression model adjusted for potential confounders, the robotic approach remained independently associated with an 89% reduction in the odds of conversion (Adjusted OR 0.11; 95% CI 0.02–0.77; p = 0.027).

### Postoperative outcomes: robotic vs. laparoscopic

The primary comparative analysis between robotic and laparoscopic approaches revealed superior recovery outcomes for the robotic platform across multiple metrics (Table 3). In the primary 1:1 propensity-matched analysis, the Robotic group demonstrated a 16.1% absolute risk reduction in 30-day readmissions compared to the laparoscopic group (p = 0.025). Furthermore, the robotic group had a significantly lower risk of serious complications (Clavien–Dindo ≥ III), with a risk difference of -6.5% (p = 0.037). Regarding recovery time, while the initial 1:1 matching showed a trend toward reduced length of stay (p = 0.063), the robust sensitivity analysis (1:4 matching) incorporating a larger control cohort confirmed a statistically significant reduction in hospital stay for the robotic group (p = 0.049).

**Table 3:** Comparative outcomes (robotic vs. laparoscopic)

Outcomes	Adjusted OR / Coef (95% CI)	Reg P–val	PSM 1:1 Diff (ATET) [95% CI]	PSM 1:1 P val	Sens. (1:4) P–val
Length of stay (days)	–2.87 (–5.3, –0.4)	0.021	–1.22 [–2.5, 0.1]	0.063	0.049
Operative time (min)	–3.8 (–47.8, 40.2)	0.865	–37.7 [–168, 92]	0.572	0.612
Conversion to open	0.11 (0.02, 0.77)	0.027	–3.2% [–7.5, 1.0]	0.140	0.114
30-day complications	0.61 (0.31, 1.19)	0.150	–12.9% [–27.9, 2.1]	0.092	0.124
30-day serious complications	N/A (zero events)	N/A	–6.5% [–12.5, –0.4]	0.037	0.041
30-day readmission	0.58 (0.28, 1.18)	0.136	–16.1% [–30.2, –2.0]	0.025	0.032
30-day reoperation	N/A (zero events)	N/A	–1.6% [–4.7, 1.5]	0.313	0.288

Data are presented as adjusted odds ratios (OR) or coefficients (Coef) with 95% confidence intervals (CI) derived from multivariable regression models. “PSM 1:1 Diff” represents the average treatment effect on the treated (ATET) utilizing 1:1 propensity score matching. Sensitivity analysis (“Sens.”) P-values are derived from 1:4 matching for robustness

Model Adjustments: All regression and PSM models were adjusted for age, sex, BMI, ASA class, comorbidities, disease duration, disease behavior, medications, prior surgery, and concomitant procedures

*ASA*, American Society of Anesthesiologists; *ATET*, average treatment effect on the treated; *BMI*, body mass index; *CI*, confidence interval; *LOS*, length of stay; *PSM*, propensity score matching; *Reg*, regression

### Postoperative outcomes: robotic vs. open

The comparison between robotic and open surgery highlighted the safety profile of the robotic platform. No patients in the robotic group required reoperation (0%) or suffered a serious complication (0%), compared to 4.7% and 7.9% in the open group, respectively.

The sensitivity analysis utilizing 1:4 propensity score matching revealed a highly statistically significant benefit in recovery. When compared against a larger matched cohort of open patients, robotic surgery was associated with a significant reduction in length of stay (Coefficient -3.44 days; p < 0.001). This confirms that the robotic platform offers a substantial recovery advantage over open surgery, a finding that was underestimated in the primary 1:1 analysis due to sample size constraints (Table 4).

**Table 4:** Comparative outcomes (robotic vs. open)

Outcomes	Adjusted OR / Coef (95% CI)	Reg P val	PSM 1:1 Diff (ATET) [95% CI]	PSM 1:1 P val	Sens. (1:4) P val
Length of stay (days)	–12.4 (–38.9, 14.2)	0.363	–3.44 [–10.8, 3.9]	0.357	< 0.001
Operative time (min)	–14.6 (–105.5, 76.3)	0.753	–43.2 [–177, 91]	0.529	0.491
30-d ay complications	0.61 (0.31, 1.19)	0.147	–6.5% [–22.2, 9.3]	0.423	0.388
Serious complications	N/A (zero events)	N/A	–4.8% [–10.2, 0.5]	0.080	0.076
Readmission	0.51 (0.25, 1.06)	0.070	–14.5% [–31.6, 2.6]	0.096	0.088
Reoperation	N/A (zero events)	N/A	–6.5% [–13.3, 0.4]	0.067	0.062

Data are presented as adjusted odds ratios (OR) or coefficients (Coef) with 95% confidence intervals (CI) derived from multivariable regression models. “PSM 1:1 Diff” represents the average treatment effect on the treated (ATET) utilizing 1:1 propensity score matching. Sensitivity analysis (“Sens.”) P values are derived from 1:4 matching for robustness

Model Adjustments: All regression and PSM models were adjusted for age, sex, BMI, ASA class, comorbidities, disease duration, disease behavior, medications, prior surgery, and concomitant procedures

*ASA*, American Society of Anesthesiologists; *ATET*, average treatment effect on the treated; *BMI*, body mass index; *CI*, confidence interval; *LOS*, length of stay; *PSM*, propensity score matching; *Reg*, regression

### Sensitivity analysis (G-computation)

G-computation analysis **(**Table 5) reinforced the robustness of the primary findings. The adjusted risk of conversion to open surgery was 1.7% for the robotic approach versus 14.8% for laparoscopy (p < 0.001). Regarding recovery, the analysis revealed that robotic surgery had the shortest adjusted mean length of stay compared to laparoscopic (p = 0.003) and open (p < 0.001) approaches, confirming the recovery benefit observed in the sensitivity analysis.

**Table 5:** Adjusted outcomes (G-computation)

Outcomes	Robotic	Laparoscopic	Open	*P* value (rob vs. lap)	*P* value (rob vs. open)	*P* value (lap vs. open)
30-day complications	21.6%	30.0%	30.7%	0.12	0.10	0.82
Serious complications	N/A*	6.8%	7.3%	N/A	N/A	0.72
Readmission	17.4%	26.2%	28.7%	0.08	0.034	0.32
Reoperation	N/A*	4.5%	0.8%	N/A	N/A	0.99
Conversion to open	1.7%	14.8%	N/A	< 0.001	N/A	N/A
Length of stay (days)†	9.7	13.7	25.1	0.003	< 0.001	< 0.001
Operative time (min)	227	236	219	0.62	0.70	0.50

^*^Due to 0 events in the robotic group, the G-computation model could not predict a valid adjusted risk or P value for serious complications or reoperations for that group

## Discussion

This contemporary propensity-matched analysis indicates that robotic ileocecal resection confers clear advantages over both conventional laparoscopy and open surgery in Crohn’s disease. Compared with laparoscopy, the robotic platform significantly improved postoperative recovery, with a 16.1% absolute risk reduction in 30-day readmissions (p = 0.025) and a markedly lower conversion rate (adjusted OR 0.11, p = 0.027). The robotic cohort also demonstrated an excellent safety profile, with zero serious complications, corresponding to a significant risk reduction compared with laparoscopy (risk difference − 6.5%, p = 0.037), and showed a trend toward improved outcomes over open surgery, reflected by a 14.5% reduction in readmission risk (p = 0.096). These benefits were achieved without sacrificing operative efficiency, as adjusted operative times were comparable between robotic and laparoscopic approaches (p = 0.572), thereby supporting robotic ileocecal resection as a preferred minimally invasive modality for Crohn’s disease.

The principal finding of this analysis is the pronounced “anti-conversion” effect associated with the robotic platform in Crohn’s disease surgery, with conversion rates of 1.6% for robotics versus 15.2% for laparoscopy. This translates into an 89% reduction in the odds of conversion (adjusted OR 0.11) after adjustment for confounding variables. This striking difference likely reflects the robot’s capacity to mitigate the specific technical challenges of Crohn’s inflammatory pathology, including adhesions from prior procedures, thickened mesentery, and inflammatory masses, which are well-established predictors of laparoscopic conversion [16]. In such settings, standard laparoscopic instruments frequently prove inadequate, with conversion reported in up to 34% of cases with dense adhesions [17], whereas the robotic system facilitates more precise dissection through inflamed tissue planes, effectively providing a “rescue” capability that permits minimally invasive completion of complex cases. In addition, the robotic platform allows more reliable hemostasis during mesentery dissection, thanks to the simultaneous use of bipolar and radiofrequency energy (robotic Ligasure, Medtronic, Minneapolis, MN, USA). These observations are concordant with the broader literature, including a recent meta-analysis identifying the robotic approach as an independent protective factor against conversion (OR 0.44) [18] and large-scale cohorts reporting significantly lower conversion rates with robotics compared with laparoscopy [19]. Given that conversion is associated with increased postoperative morbidity and prolonged length of stay in Crohn’s disease surgery, the ability of the robotic platform to preserve a minimally invasive approach represents a clinically meaningful advance for this high-risk population [20].

The present analysis identifies a notable safety and recovery advantage of the robotic approach, characterized by a significantly reduced 30-day readmission rate and an observed absence of serious postoperative complications in the robotic cohort. The reduction in readmissions (17.4% vs. 26.2% unadjusted, corresponding to a 16.1% absolute risk reduction in the matched analysis) likely reflects a multifactorial benefit, in which gentler tissue handling and enhanced precision during dissection, collectively minimize surgical trauma and postoperative inflammatory response, thereby potentially reducing the incidence of ileus, dehydration, and infectious complications that commonly drive readmission in Crohn’s disease. The “zero-complication” finding, particularly the absence of anastomotic leakage and reoperation, should be interpreted with appropriate caution given the sample size; however, when contrasted with the serious complication rates observed in both the laparoscopic (6.8%) and open (7.9%) groups, it provides a compelling safety signal that supports the hypothesis of a genuine risk reduction rather than a purely stochastic effect, as shown in a recent meta-analysis [21]. Moreover, when benchmarked against laparoscopic and open surgery in a propensity-matched 1:4 sensitivity analysis, the robotic approach was associated with a significantly shorter length of stay, underscoring that the minimally invasive benefits of the robotic platform are preserved, and may even be accentuated, in a cohort that typically includes more complex and high-risk Crohn’s disease cases, as shown in recent publications [22–25].

This analysis also challenges the prevailing perception that robotic surgery compromises operative efficiency. While unadjusted operative times favored laparoscopy (170 vs. 205 min), multivariable adjustment for case complexity, including concomitant procedures and disease behavior, eliminated this difference (p = 0.572). These results suggest that procedural complexity, rather than the robotic platform per se, primarily accounts for duration differences in Crohn’s disease surgery. Furthermore, as one of the initial reports evaluating the Hugo™ RAS system for ileocecal resection in this setting, these findings affirm the platform’s feasibility and safety profile, with outcomes equivalent or superior to conventional laparoscopic and open techniques [26, 27]. With accumulating institutional experience, particularly regarding docking and console optimization along the learning curve, additional reductions in operative time are anticipated, potentially amplifying the robotic approach’s efficiency advantages.

This study has several limitations inherent to its retrospective design and non-randomized nature. First, the sample size of the robotic group was relatively small compared to the extensive laparoscopic and open cohorts, reflecting the more recent adoption of the platform at our institution. While sensitivity analyses utilizing 1:4 propensity score matching were employed to increase effective sample size, this imbalance inherently limits the statistical power to detect differences in very rare adverse events. Second, despite the utilization of robust matching techniques and G-computation to balance baseline characteristics, including surgical complexity, residual confounding cannot be excluded. Third, the complete absence of serious complications and reoperations in the robotic group, while clinically favorable, presented statistical challenges that prevented the calculation of adjusted odds ratios for these specific endpoints in multivariable regression models. Finally, as a single-center experience from a high-volume tertiary referral unit utilizing the Hugo™ RAS system, our findings regarding operative efficiency and safety may not be immediately generalizable to lower-volume centers or surgeons utilizing different robotic platforms.

## Conclusion

In conclusion, robotic ileocecal resection utilizing the Hugo™ RAS system represents a safe and effective alternative to conventional laparoscopy and open surgery. The robotic platform is associated with a marked reduction in conversion rates and hospital readmissions, alongside a superior safety profile evidenced by a significantly lower risk of serious complications compared to laparoscopy. Additionally, sensitivity analyses demonstrate a significant benefit in reduced length of hospital stay compared to both conventional approaches. These clinical advantages are achieved without a significant increase in adjusted operative time, supporting the continued adoption of robotic technology for complex Crohn’s disease resections.
